# Arousal-informed neuronal avalanches operate near criticality

**DOI:** 10.64898/2026.10.12.757995

**Published:** 2026-10-13

**Authors:** Cédric Stefens, Hardik Rajpal, Joe S. Canzano, Meghdad Saeedian, Michael G. Kareithi, Henrik Jeldtoft Jensen, Mauricio Barahona, Spencer LaVere Smith, Simon R. Schultz

## Abstract

Cortical networks are thought to operate near a “critical point” that optimizes information processing, but this has been difficult to test at single-cell resolution across large brain areas, as narrow fields of view miss overlapping neural avalanches, causing subcritical estimates, Using mesoscopic two-photon calcium imaging in awake mouse visual cortex, we recorded thousands of neurons and applied convolutional non-negative matrix factorization to decompose population activity into distinct, overlapping spatiotemporal avalanches. Rather than occupying a single dynamical state, we find that the cortex hosts multiple overlapping subnetworks distributed across a phase diagram at varying distances from the critical point. Arousal-linked avalanches propagate widely and operate nearest the critical point, showing optimized dynamic range, minimized algorithmic complexity, and maximized temporal entropy. Our results reveal how the awake brain tunes parallel near-critical subnetworks to support robust sensory computation.

